# Diagnoses given in specialist health care to Norwegian-born children with one immigrant parent. A register-based study

**DOI:** 10.1177/14034948251344527

**Published:** 2025-06-12

**Authors:** Marte K. R. Kjøllesdal, Ylva Helland, Thor Indseth

**Affiliations:** 1 Norwegian University of Life Sciences, Department of Public Health Science, Ås, Norway; 2 Norwegian Institute of Public Health, Health Services Research, Oslo, Norway

**Keywords:** Migration, childhood, socioeconomic position, specialist health care, diagnoses

## Abstract

**Objective:**

To compare the risk of receiving diagnoses of somatic health problems in specialist health care among Norwegian-born children with one immigrant parent to children of Norwegian background.

**Methods:**

Data from Medical Birth Registry of Norway, Norwegian Patient Registry and Statistics Norway were linked. All children born in Norway to two Norwegian-born parents or to one Norwegian-born parent and one immigrant parent, aged 0–10 years between 2008 and 2018 were included. Diagnostic categories of infections, non-infectious medical conditions and non-infectious neurological conditions were included from 2008 onwards. Hazards of diagnoses by immigrant background were assessed by Cox regressions adjusted for sex and birth year, and additionally for parental education and household income.

**Results:**

Compared with children with Norwegian background, children with an immigrant mother had lower hazards of receiving a diagnosis of any somatic condition (hazard ratio 0.97, 95% confidence interval (CI) 0.95, 0.98), infections in total (hazard ratio 0.93, 95% CI 0.91, 0.95), total non-infectious medical conditions (hazard ratio 0.97, 95% CI 0.95, 0.98) and of any neurological condition (hazard ratio 0.89, 95% CI 0.86, 0.93). There were variations for individual diagnosis groups and by the immigrant parent being a mother or a father, and by parental region of origin. Adjustments for socioeconomic variables made negligible changes to estimates.

**Conclusions:**

Children of one immigrant parent do not have overall worse health than children with Norwegian background. Lower hazards of diagnoses of many conditions among those with an immigrant mother warrants further research into possible underutilization of services and barriers to use.

## Introduction

The number of children born in Europe to immigrant parents is on the rise. The knowledge about the health in this group is still limited, but existing studies suggest that health among children varies with parental immigrant background [1–3]. Certain forms of barriers to health care and health information might be more common for parents with immigrant background, such as low proficiency in the language of the host country, low health literacy and limited knowledge about health systems [4]. For their children, this can be a barrier to timely and correct care. In addition, perceptions of health and disease and when to seek health care may vary with cultural background [4]. Immigrants are a highly heterogenous group with regard to cultural background, language skills, education, work opportunities and health. However, many, especially immigrants from low- and middle-income countries, experience a disproportionate burden of disease [5], and disadvantaged socioeconomic positions (SEPs), having little education, low household income and poor housing and neighbourhood conditions. For children of immigrants, growing up in these conditions could have negative influences on health [6–8]. Although SEP is important for the health of children, most studies find that indicators of socioeconomic condition do not explain differences between children with immigrant parents and children with native parents [2]. An accumulation of factors, related to both socioeconomic and demographic conditions which, when operating together contribute to an increased vulnerability, has been suggested as an important mechanism [9].

The upbringing of children growing up in exogamous families (with one immigrant and one native born parent) will be influenced by factors related to one parent being an immigrant, such as cultural perceptions about health and lifestyle other than in the native population [10]. But it will also have resources related to a native parent available, such as knowing the health care system and the language. Moreover, immigrants married to a native tend to have higher SEP than other immigrants. Explanations include a selection of immigrants from higher SEP marrying natives, or natives with high SEP finding a spouse when abroad for higher education or work, or a spillover of information and networks between natives and immigrants through marriage [11]. Children and youth raised in exogamous families are shown to have higher risk than their peers of being treated for psychological conditions [10,12]. This is likely to reflect struggles related to trying to fit into multiple identities, parental stress related to migration experiences and tensions of intermarriages, and, for some groups, discrimination, although some also experience positive resilience from growing up with two cultures [10]. The use of health services for these conditions might be influenced by cultural perceptions of mental health and related stigma. Differences between children in exogamous and in native families regarding somatic conditions are less clear [10,12], and more likely to reflect differences in SEP and resources or barriers to health care, including system knowledge and language proficiency.

The immigrant population residing in Norway during the study period was heterogeneous, consisting of both labour migrants and refugees. In addition to immigrants from Scandinavian countries, Norway experienced an influx of labour migration in the 1970s from Pakistan, Turkey and Morocco, and later during the 2000s from countries such as Poland, Lithuania, Thailand and the Philippines. Refugees came from Chile, Vietnam and Iran in the 1970s and 1980s, from former Yugoslavia in the 1990s and from Somalia, Sri Lanka, Iraq, Eritrea and Syria in the 2000s. During the study period a significant proportion of immigrants in Norway was relatively new, with about one in four having lived in the country for less than five years [13]. We have previously reported differences in risk of receiving diagnoses of somatic conditions in specialist health care among Norwegian-born children with two immigrant parents compared with children with Norwegian background [1]. Children of immigrants had higher hazards than children with Norwegian parents of a diagnosis of several types of infections, obesity, nutrition-related disorders, and skin diseases, and lower risk of a diagnosis of lower respiratory tract infections and disease, diseases of the circulatory system, immune system disorders and headache conditions. Our hypothesis is first that these differences can partly be explained by underuse of services and barriers to care related to parental knowledge of the system and language proficiency. If such barriers are of importance, the patterns of diagnoses among children with one immigrant parent should be somewhere between that of children with two immigrant parents and those with Norwegian background. Further, as the mother is the caregiver most often seeking health care for their children, the differences from children with a Norwegian background would be smaller among children with a Norwegian-born mother and an immigrant father than vice versa. Second, we hypothesize that higher risk of some conditions might be related to low SEP in immigrant families, and that because of higher SEP in many exogamous families, children with one immigrant parent might be more like children with Norwegian background also regarding risk of receiving these diagnoses. To test these hypotheses, the aim of this article was 1) to compare the risk of receiving diagnoses of somatic health conditions in specialist health care among Norwegian-born children with one immigrant and one Norwegian-born parent with children with two Norwegian-born parents, 2) to assess any differences in risk of diagnoses by having an immigrant mother versus an immigrant father, and 3) to assess the importance of SEP (measured as parental education and household income) in the differences we might find. Recognizing the differences between groups of immigrants, we have carried out all analyses both in the whole sample and by parental regional background. In the discussion, we will compare our results with previously published results on children with two immigrant parents.

## Methods

### Study population

All children born in Norway to two Norwegian-born parents or to one Norwegian-born parent and one immigrant parent, as registered in the Norwegian Medical Birth Register and who were 0–10 years of age between 2008 and 2018 (i.e. children born in 1998–2017) were included (*N*=1,019,842). We excluded children both of whose parents had no registered country of birth (*n*=13,413), who were registered as emigrated (data on emigration year not available) (*n*=7033), stillborn (*n*=22), dead prior to 2008 (*n*=1940) and children with missing information on both parents’ educational level (*n*=275) or on household income in any of the years they were included in analyses (*n*=6792), leaving a sample of 990,637 children.

Data from the Medical Birth Registry of Norway (MBRN) were linked to data from the Norwegian Patient Registry (NPR) and to data from Statistics Norway on immigrant background, parental country of origin and educational level and household income, using personal identification number. Every child born in Norway is registered in MBRN, with identification of parents [14]. The NPR contains ICD-10 (International Classification of Disease) diagnoses given in specialist health care (both inpatient and outpatient) from 2008 [15].

### Variables

We included 37 diagnostic categories from three domains of physical health: infections, non-infectious medical conditions, and non-infectious neurological conditions, registered from 2008 (start of NPR) to 2018 (ICD-10 codes in Supplemental material Table I online). Children who had a specific diagnosis registered in NPR at least once during the specified age and time frame were classified as having the respective diagnosis. An additional variable was made to indicate whether they had received any of the 37 included diagnoses. Among children with one immigrant parent, region of origin was classified according to national standards [16], based on information about parents’ country of birth (if different, mother’s): ‘EU/European Economic Area (EEA), Oceania, United States of America (USA) and Canada’ (hereafter called EU/EEA), ‘Europe outside the EU/EEA’, ‘Asia’, ‘Africa’ and ‘Latin America’. The countries represented in each region are shown in Supplemental Table II.

The highest attained education of either parent registered in 2017 was recoded from nine to four categories: ‘primary school’ (started or completed/⩽9 years), ‘upper secondary school’ (12 years), ‘university/university college, lower’ (completed a university/university college education of ⩽4 years) and ‘university/university college, higher’ (completed a university/university college education of >4 years). In Statistics Norway, annual household income (in Norwegian kroner) after tax was noted and divided by number of consumption units (EU scale) in the household. In analyses, household income was included as a continuous covariate varying over each included year. In addition, quartiles of household income were made based on the average income for the years under follow-up. The average household income by consumption units in each quartile was: Quartile 1: NOK204,837, Quartile 2: NOK273,977, Quartile 3: NOK326,280, Quartile 4: NOK480,748. The percentages receiving each diagnosis according to immigrant background and region of origin were reported. Chi square tests were applied to compare the percentages receiving each diagnosis by each regional background compared with those with Norwegian background. In Cox proportional hazard regressions, hazard ratio with 95% confidence intervals (CIs) for each diagnosis were calculated for children with an immigrant mother or an immigrant father, as well as by region of origin, compared with children of Norwegian background.

Analyses were adjusted for 1) sex and birth year and 2) additionally for parental education and household income. Children were followed from 2008, or year of birth (if later than 2008), until year of diagnosis, year of death, year of reaching 10 years of age (if earlier than 2018) or 2018. The number of years of follow-up in analyses thus varied by year of birth. Analyses were performed using STATA 18 (StataCorp LLC, College Station, TX, USA). We carried out two sensitivity analyses, one including those with missing parental country of birth and one including those with missing information on household income, both comparing children with one immigrant parent with those with Norwegian. For both, there were negligible differences in results. We report unadjusted numbers, in addition to adjusted numbers, in the results section. The purpose of this is to identify potential target groups for interventions.

## Results

The sample consisted of 990,637 children, of which 85.4 % had two Norwegian-born parents, 7.9% an immigrant mother and 6.7% an immigrant father (Table I). Of those with one immigrant parent, the majority had a parent from EU/EEA. A higher proportion of children with one immigrant parent (except EU/EEA) had parents with primary education than children with Norwegian background, but also higher proportions of parents with high higher education (except Asia) (Table I). A higher proportion of children with one immigrant parent (except EU/EEA) than others had low household income. The proportions having received the different diagnosis by having an immigrant mother or father and by parental region of origin are shown in Supplemental Table IIIa and IIIb.

**Table I.** Characteristics of the sample.

	Norwegian background *n*=846,182 (85.4%)	Immigrant mother *n*=78,497 (7.9%)	Immigrant father *n*=65,958 (6.7%)	One immigrant parent, total *n*=144,455 (14.6%)	EU, EEA, Oceania, USA, Canada^a^ *n*=76,966 (7.8%)	Europe outside EU/EEA^a^ *n*=8290 (0.8%)	Asia^a^ *n*=37 815 (3.8%)	Africa^a^ *n*=9372 (1.0%)	Latin America^a^ *n*=12,012 (1.2%)
Girls (%)	48.7	48.4	48.7	48.5	48.7	48.5	48.2	49.1	48.1
Parental education (%)									
Primary	4.7	7.0	7.6	7.3	4.3	7.9	12.7	9.0	8.0
Upper secondary	33.2	28.9	24.1	26.7	22.2	26.7	34.7	25.7	31.1
Higher, low	42.3	35.5	39.7	37.4	39.3	32.6	34.2	40.9	36.0
Higher, high	19.8	28.7	28.6	28.6	34.3	32.9	18.4	24.4	25.0
Household income (%)									
Quartile 1	24.1	30.1	31.4	29.0	31.4	22.9	39.4	43.7	36.3
Quartile 2	25.4	22.7	23.2	22.3	23.2	21.6	23.8	24.9	24.7
Quartile 3	25.6	21.8	21.6	21.9	21.6	23.9	18.7	17.8	20.6
Quartile 4	24.9	25.4	23.8	26.7	23.8	31.6	18.1	14.2	18.5

a Region by either immigrant mother or father.

EU/EEA=EU/European Economic Area (EEA), Oceania, United States of America (USA) and Canada.

The hazard of a diagnosis of any somatic condition was slightly lower among children with one immigrant parent than among children with Norwegian background, but not in the fully adjusted model (Table II). Children with an immigrant mother had lower, and children with an immigrant father higher, hazard of receiving a diagnosis of any somatic condition (Table II). Children with a parent from EU/EEA and Asia had lower, whereas children with a parent from Europe except EU/EEA, Africa and Latin America had higher, hazard of any diagnosis of a somatic condition compared with children with Norwegian background (Table III).

**Table II.** Hazard ratio (95% confidence interval) for diagnoses given in secondary and tertiary health care between 2008 and 2018 among children 0–10 years old having one immigrant parent. Reference: Norwegian background. From Cox regressions, adjusted for year of birth and sex (model 1) and additionally for parental education and household income (model 2).

	Norwegian-born to one immigrant parent, total		Immigrant mother		Immigrant father	
	Model 1	Model 2	Model 1	Model 2	Model 1	Model 2
**Any somatic condition**	0.98 (0.97, 0.99)	0.99 (0.99, 1.00)	0.97 (0.95, 0.98)	0.97 (0.97, 0.98)	1.01 (0.99, 1.02)	1.02 (1.01, 1.02)
**Infections**						
Infections total	0.97 (0.95, 0.98)	0.99 (0.98, 0.99)	0.93 (0.91, 0.95)	0.95 (0.94, 0.96)	1.01 (0.98, 1.03)	1.02 (1.02, 1.03)
Intestinal infectious diseases	0.99 (0.96, 1.03)	1.00 (0.98, 1.01)	0.98 (0.94, 1.03)	0.98 (0.97, 1.00)	1.00 (0.95, 1.05)	1.01 (0.99, 1.03)
Tuberculosis	6.35 (3.53, 11.43)	7.23 (5.89, 8.88)	9.07 (4.8, 16.92)	10.03 (8.06, 12.48)	2.88 (1.08, 7.64)	3.59 (2.61, 4.95)
Other bacterial infections and sexually transmitted diseases	0.92 (0.86, 0.98)	0.93 (0.91, 0.95)	0.90 (0.83, 0.98)	0.91 (0.88, 0.93)	0.94 (0.86, 1.02)	0.95 (0.92, 0.98)
Viral infections	0.99 (0.96, 1.03)	1.01 (1.00, 1.02)	0.96 (0.92, 1.00)	0.98 (0.96, 0.99)	1.03 (0.98, 1.08)	1.05 (1.04, 1.07)
Fungal and parasitic infections	1.20 (1.08, 1.33)	1.23 (1.18, 1.27)	1.21 (1.06, 1.38)	1.27 (1.21, 1.33)	1.18 (1.02, 1.37)	1.17 (1.11, 1.23)
Influenza and other acute lower respiratory tract infections	0.84 (0.82, 0.87)	0.86 (0.85, 0.87)	0.78 (0.75, 0.81)	0.80 (0.78, 0.81)	0.93 (0.89, 0.97)	0.94 (0.92, 0.95)
Infections of the skin and subcutaneous tissue	1.29 (1.24, 1.35)	1.30 (1.28, 1.32)	1.26 (1.19, 1.34)	1.26 (1.24, 1.29)	1.33 (1.25, 1.42)	1.33 (1.30, 1.36)
Infections of the musculoskeletal system and soft tissue	0.89 (0.83, 0.96)	0.90 (0.88, 0.93)	0.87 (0.79, 0.96)	0.89 (0.86, 0.92)	0.92 (0.83, 1.02)	0.92 (0.89, 0.96)
Urinary tract infections	0.93 (0.87, 1.00)	0.94 (0.91, 0.96)	0.91 (0.83, 1.00)	0.92 (0.89, 0.95)	0.96 (0.87, 1.05)	0.96 (0.92, 0.99)
Genital infections	1.08 (0.94, 1.23)	1.10 (1.05, 1.15)	1.10 (0.93, 1.31)	1.11 (1.04, 1.18)	1.04 (0.86, 1.26)	1.09 (1.02, 1.16)
Infections of the CNS	0.78 (0.66, 0.91)	0.77 (0.72, 0.81)	0.71 (0.57, 0.88)	0.69 (0.64, 0.75)	0.86 (0.69, 1.08)	0.86 (0.79, 0.93)
**Non-infectious medical conditions**						
Any non-infectious medical condition	0.98 (0.97, 0.99)	0.99 (0.98, 0.99)	0.97 (0.95, 0.98)	0.97 (0.96, 0.98)	1.00 (0.98, 1.02)	1.01 (1.01, 1.02)
Malignant neoplasms (outside CNS)	0.88 (0.73, 1.06)	0.91 (0.85, 0.97)	0.78 (0.60, 1.01)	0.80 (0.73, 0.88)	1.00 (0.78, 1.29)	1.04 (0.95, 1.13)
Benign neoplasms (outside CNS)	0.99 (0.95, 1.03)	0.97 (0.96, 0.99)	0.98 (0.93, 1.04)	0.96 (0.94, 0.98)	1.00 (0.94, 1.06)	0.99 (0.97, 1.01)
Blood diseases	1.06 (0.99, 1.13)	1.02 (0.99, 1.04)	1.03 (0.95, 1.13)	0.99 (0.95, 1.02)	1.10 (1.00, 1.20)	1.06 (1.02, 1.10)
Immune system disorders	0.73 (0.59, 0.89)	0.76 (1.10, 1.11)	0.66 (0.50, 0.86)	0.68 (0.62, 0.76)	0.81 (0.62, 1.07)	0.85 (0.77, 0.94)
Endocrine disorders	0.96 (0.90, 1.02)	0.97 (0.95, 0.99)	0.89 (0.81, 0.96)	0.88 (0.85, 0.90)	1.05 (0.97, 1.15)	1.08 (1.05, 1.11)
Malnutrition and problems with eating and feeding	1.08 (1.02, 1.14)	1.07 (1.05, 1.09)	1.08 (1.00, 1.16)	1.08 (1.05, 1.11)	1.08 (1.00, 1.17)	1.06 (1.03, 1.10)
Other nutritional deficiencies	1.27 (1.13, 1.43)	1.24 (1.19, 1.30)	1.16 (0.99, 1.36)	1.15 (1.09, 1.23)	1.40 (1.20, 1.65)	1.35 (1.27, 1.44)
Obesity and other hyperalimentation	1.21 (1.11, 1.33)	1.21 (1.18, 01.25)	1.08 (0.95, 1.22)	1.04 (1.00, 1.08)	1.38 (1.22, 1.56)	1.43 (1.38, 1.49)
Metabolic disorders	0.80 (0.69, 0.92)	0.80 (0.76, 0.84)	0.72 (0.59, 0.88)	0.71 (0.67, 0.77)	0.90 (0.74, 1.09)	0.90 (0.84, 0.96)
Visual impairment/blindness	0.77 (0.64, 0.92)	0.80 (0.75, 0.85)	0.68 (0.53, 0.87)	0.72 (0.66, 0.78)	0.88 (0.69, 1.11)	0.89 (0.82, 0.97)
Hearing impairment/deafness	0.82 (0.79, 0.86)	0.83 (0.81, 0.84)	0.81 (0.76, 0.86)	0.81 (0.79, 0.82)	0.84 (0.79, 0.90)	0.85 (0.83, 0.87)
Diseases of the circulatory system outside of the CNS	0.89 (0.82, 0.97)	0.87 (0.85, 0.90)	0.89 (0.80, 0.99)	0.85 (0.82, 0.89)	0.90 (0.80, 1.01)	0.90 (0.86, 0.93)
Chronic lower respiratory disease (including asthma)	0.88 (0.86, 0.90)	0.90 (0.90, 0.91)	0.82 (0.85, 0.87)	0.87 (0.86, 0.88)	0.91 (0.88, 0.94)	0.96 (0.95, 0.97)
Diseases of the digestive system	0.89 (0.87, 0.91)	0.90 (0.90, 0.91)	0.84 (0.81, 0.86)	0.87 (0.86, 0.88)	0.96 (0.93, 0.99)	0.94 (0.93, 0.95)
Diseases of the skin and soft tissue	1.18 (1.16, 1.20)	1.19 (1.18, 1.20)	1.22 (1.20, 1.26)	1.23 (1.22, 1.24)	1.12 (1.09, 1.15)	1.14 (1.13, 1.15)
Diseases of the musculoskeletal system and connective tissue	0.97 (0.94, 0.99)	0.97 (0.96, 0.98)	0.90 (0.87, 0.94)	0.91 (0.90, 0.92)	1.04 (1.00, 1.08)	1.04 (1.03, 1.06)
Urinary tract diseases	0.87 (0.83, 0.92)	0.88 (0.87, 0.90)	0.83 (0.77, 0.89)	0.84 (0.82, 0.86)	0.92 (0.86, 0.99)	0.93 (0.91, 0.96)
Genital diseases and disorders of breast	1.04 (1.00, 1.08)	1.04 (1.03, 1.05)	1.01 (0.96, 1.06)	1.00 (0.99, 1.02)	1.08 (1.02, 1.14)	1.09 (1.07, 1.11)
**Non-infectious neurological conditions**						
Neurological conditions total	0.96 (0.93, 0.99)	0.97 (0.96, 0.98)	0.89 (0.86, 0.93)	0.91 (0.90, 0.92)	1.04 (0.99, 1.08)	1.04 (1.03, 1.06)
Sleeping disorders	0.96 (0.91, 1.01)	0.99 (0.97, 1.01)	0.87 (0.81, 0.93)	0.91 (0.88, 0.93)	1.08 (1.00, 1.15)	1.10 (1.07, 1.13)
Neoplasms of the CNS (malignant and benign)	0.88 (0.65, 1.20)	0.89 (0.80, 0.99)	0.95 (0.65, 1.40)	0.99 (0.87, 1.13)	0.80 (0.51, 1.26)	0.76 (0.65, 0.90)
Cerebrovascular diseases	0.92 (0.69, 1.22)	0.93 (0.84, 1.03)	1.02 (0.71, 1.45)	1.02 (0.90, 1.16)	0.81 (0.52, 1.24)	0.82 (0.70, 0.96)
Epilepsy	0.98 (0.91, 1.05)	0.99 (0.97, 1.02)	0.93 (0.85, 1.02)	0.96 (0.93, 0.99)	1.03 (0.94, 1.14)	1.03 (1.00, 1.07)
Headache conditions (including migraine)	0.85 (0.80, 0.90)	0.86 (0.84, 0.88)	0.78 (0.72, 0.85)	0.80 (0.78, 0.87)	0.93 (0.86, 1.01)	0.94 (0.91, 0.96)
Cerebral palsy	0.91 (0.78, 1.06)	0.90 (0.67, 0.82)	0.84 (0.68, 1.03)	0.80 (0.74, 0.87)	1.00 (0.80, 1.24)	1.02 (0.84, 1.10)
Hydrocephalus	0.77 (0.59, 0.99)	0.74 (0.67, 0.82)	0.80 (0.57, 1.11)	0.83 (0.74, 0.94)	0.73 (0.50, 1.06)	0.64 (0.55, 0.74)
Other disorders of the nervous system	1.12 (1.07, 1.19)	1.14 (1.11, 1.16)	1.08 (1.01, 1.16)	1.09 (1.06, 1.12)	1.18 (1.09, 1.27)	1.19 (1.16, 1.22)

CNS: central nervous system.

**Table III.** Hazard ratio (95% confidence interval) for diagnoses given in secondary and tertiary health care between 2008 and 2018 among children 0–10 years old having one immigrant parent, by parental region of origin. Reference: Norwegian background. From Cox regressions, adjusted for year of birth and sex (model 1) and additionally for parental education and household income (model 2).

	EU, EEA, USA, Canada, Oceania		Europe except EU/EEA		Asia		Africa		Latin America	
	Model 1	Model 2	Model 1	Model 2	Model 1	Model 2	Model 1	Model 2	Model 1	Model 2
**Any somatic condition**	0.97 (0.96, 0.99)	0.99 (0.99, 1.00)	1.04 (1.00, 1.08)	1.07 (1.05, 1.08)	0.95 (0.93, 0.97)	0.93 (0.93 0.94)	0.95 (0.93, 0.97)	1.06 (1.04, 1.07)	1.05 (1.01, 1.09)	1.07 (1.06, 1.08)
**Infections**										
Infections total	0.95 (0.93, 0.97)	0.98 (0.97, 0.99)	1.02 (0.96, 1.08)	1.06 (1.03, 1.08)	0.93 (0.90, 0.96)	0.93 (0.91, 0.94)	1.07 (1.01, 1.14)	1.08 (1.06, 1.11)	1.08 (1.04, 1.14)	1.09 (1.07, 1.11)
Intestinal infectious diseases	0.90 (0.86, 0.95)	0.92 (0.91, 0.94)	0.98 (0.86, 1.12)	0.96 (0.91, 1.00)	1.02 (0.96, 1.09)	0.99 (0.97, 1.02)	1.08 (0.96, 1.22)	1.06 (1.01, 1.11)	1.35 (1.23, 1.49)	1.38 (1.33, 1.42)
Tuberculosis	-	-	-	-	11.92 (5.85, 24.32)	13.32 (10.38, 17.09)	-	-	-	-
Other bacterial infections and sexually transmitted diseases	0.93 (0.86, 1.02)	0.99 (0.96, 1.02)	0.87 (0.68, 1.11)	0.91 (0.83, 0.99)	0.96 (0.86, 1.08)	0.91 (0.87, 0.95)	0.73 (0.57, 0.95)	0.70 (0.64, 0.78)	0.85 (0.69, 1.04)	0.83 (0.77, 0.89)
Viral infections	1.00 (0.96, 1.05)	1.03 (1.02, 1.05)	1.02 (0.90, 1.16)	1.05 (1.00, 1.10)	0.92 (0.86, 0.98)	0.91 (0.89, 0.93)	1.04 (0.93, 1.17)	1.06 (1.02, 1.10)	1.10 (1.00, 1.22)	1.14 (1.10, 1.18)
Fungal and parasitic infections	0.99 (0.85, 1.15)	1.05 (0.99, 1.10)	1.10 (0.73, 1.64)	1.27 (1.10, 1.46)	1.18 (0.98, 1.42)	1.07 (0.99, 1.15)	2.36 (1.81, 3.08)	2.42 (2.20, 2.66)	1.68 (1.28, 2.20)	1.84 (1.67, 2.02)
Influenza and other acute lower respiratory tract infections	0.85 (0.81, 0.88)	0.87 (0.86, 0.88)	0.77 (0.69, 0.86)	0.82 (0.78, 0.85)	0.83 (0.79, 0.88)	0.84 (0.82, 0.85)	0.90 (0.81, 1.00)	0.90 (0.86, 0.93)	0.88 (0.81, 0.96)	0.87 (0.84, 0.90)
Infections of the skin and subcutaneous tissue	1.21 (1.14, 1.28)	1.21 (1.18 (1.23)	1.41 (1.20, 1.65)	1.43 (1.35, 1.52)	1.27 (1.17, 1.38)	1.29 (1.26, 1.33)	1.77 (1.54, 2.03)	1.76 (1.68, 1.85)	1.43 (1.26, 1.63)	1.40 (1.33, 1.47)
Infections of the musculoskeletal system and soft tissue	0.93 (0.84, 1.02)	0.95 (0.91, 0.98)	0.99 (0.75, 1.29)	0.91 (0.82, 1.01)	0.81 (0.70, 0.94)	0.82 (0.78, 0.87)	0.81 (0.61, 1.08)	0.78 (0.70, 0.86)	0.96 (0.77, 1.21)	0.97 (0.90, 1.06)
Urinary tract infections	0.95 (0.87, 1.04)	0.97 (0.94, 1.00)	0.98 (0.76, 1.27)	1.00 (0.91, 1.10)	0.85 (0.75, 0.97)	0.84 (0.80, 0.88)	0.95 (0.75, 1.22)	1.00 (0.91, 1.09)	1.01 (0.82, 1.24)	0.95 (0.88, 1.03)
Genital Infections	1.03 (0.98, 1.09)	1.01 (0.95, 1.08)	1.40 (1.23, 1.59)	1.38 (1.18, 1.62)	0.87 (0.81, 0.94)	1.16 (1.08, 1.26)	1.20 (1.06, 1.37)	1.29 (1.11, 1.50)	1.25 (1.12, 1.40)	1.08 (0.94, 1.25)
Infections of the CNS	(0.91 (0.74, 1.11)	0.93 (0.87, 1.00)	0.96 (0.54, 1.70)	0.83 (0.67, 1.03)	0.57 (0.40, 0.81)	0.54 (0.47, 0.61)	0.65 (0.34, 1.25)	0.60 (0.47, 0.76)	0.59 (0.33, 1.02)	0.59 (0.48, 0.73)
**Non-infectious medical conditions**										
Any non-infectious medical condition	0.97 (0.95, 0.99)	0.99 (0.98, 1.00)	1.03 (0.98, 1.07)	1.05 (1.03, 1.07)	0.96 (0.94, 0.99)	0.94 (0.93, 0.95)	1.03 (0.99, 1.07)	1.04 (1.03, 1.06)	1.05 (1.01, 1.09)	1.05 (1.04, 1.07)
Malignant neoplasms (outside CNS)	0.83 (0.64, 1.08)	0.86 (0.78, 0.94)	1.02 (0.53, 1.97)	0.99 (0.77, 1.27)	0.79 (0.55, 1.13)	0.87 (0.77, 0.99)	1.15 (0.64, 2.09)	1.24 (1.00, 1.52)	1.16 (0.70, 1.93)	1.03 (0.85, 1.26)
Benign neoplasms (outside CNS)	1.01 (0.95, 1.06)	0.98 (0.96, 1.00)	1.12 (0.96, 1.30)	1.08 (1.02, 1.14)	0.96 (0.89, 1.04)	0.95 (0.92, 0.97)	0.79 (0.67, 0.94)	0.80 (0.75, 0.84)	1.02 (0.90, 1.16)	1.05 (1.00, 1.10)
Blood diseases	0.97 (0.88, 1.06)	0.93 (0.90, 0.97)	0.92 (0.70, 1.19)	0.79 (0.71, 0.88)	1.30 (1.16, 1.45)	1.25 (1.20, 1.30)	1.28 (1.03, 1.59)	1.21 (1.12, 1.52)	0.83 (0.66, 1.05)	0.82 (0.75, 0.89)
Immune system disorders	0.84 (0.65, 1.08)	0.93 (0.85, 1.02)	0.22 (0.05, 0.86)	0.16 (0.08, 0.29)	0.68 (0.47, 0.99)	0.69 (0.60, 0.79)	0.81 (0.40, 1.63)	0.67 (0.51, 0.89)	0.52 (0.25, 1.09)	0.46 (0.34, 0.61)
Endocrine disorders	0.97 (0.89, 1.05)	0.98 (0.95, 1.02)	0.83 (0.64, 1.07)	0.80 (0.73, 0.88)	0.91 (0.81, 1.03)	0.93 (0.89, 0.97)	1.25 (1.02, 1.52)	1.22 (1.14, 01.30)	0.96 (0.79, 1.17)	0.94 (0.88, 1.01)
Malnutrition and problems with eating and feeding	0.97 (0.90, 1.05)	1.00 (0.97, 1.03)	0.96 (0.78, 1.19)	0.96 (0.88, 1.04)	1.33 (1.21, 1.45)	1.26 (1.22, 1.31)	0.98 (0.80, 1.20)	0.90 (0.83, 0.97)	1.11 (0.94, 1.31)	1.08 (1.01, 1.15)
Other nutritional deficiencies	0.96 (0.80, 1.15)	0.98 (0.92, 1.05)	1.15 (0.73, 1.82)	1.30 (1.11, 1.53)	1.95 (1.64, 2.31)	1.81 (1.70, 1.93)	1.32 (0.87, 1.99)	1.18 (1.01, 1.39)	0.99 (0.66, 1.48)	0.93 (0.80, 1.09)
Obesity and other hyperalimentation	0.84 (0.73, 0.97)	0.96 (0.92, 1.01)	1.77 (1.32, 2.36)	1.78 (1.62, 1.96)	1.52 (1.31, 1.76)	1.24 (1.18, 1.30)	2.23 (1.76, 2.83)	2.24 (2.07, 2.42)	1.48 (1.14, 1.91)	1.36 (1.24, 1.48)
Metabolic disorders	0.88 (0.73, 1.06)	0.90 (0.34, 0.96)	0.79 (0.45, 1.39)	0.70 (0.57, 0.87)	0.71 (0.53, 0.94)	0.70 (0.63, 0.77)	0.70 (0.40, 1.23)	0.66 (0.54, 0.81)	0.66 (0.40, 1.10)	0.64 (0.53, 0.77)
Visual impairment/blindness	0.84 (0.66, 1.05)	0.92 (0.85, 1.00)	0.56 (0.25, 1.25)	0.60 (0.45, 0.87)	0.85 (0.62, 1.16)	0.81 (0.73, 0.91)	0.51 (0.23, 1.13)	0.49 (0.37, 0.66)	0.44 (0.21, 0.93)	0.40 (0.30, 0.52)
Hearing impairment/deafness	0.88 (0.83, 0.93)	0.91 (0.89, 0.93)	0.69 (0.57, 0.84)	0.73 (0.68, 0.78)	0.74 (0.68, 0.81)	0.70 (0.67, 0.72)	0.73 (0.61, 0.88)	0.73 (0.68, 0.77)	0.89 (0.78, 1.03)	0.87 (0.83, 0.92)
Diseases of the circulatory system outside of the CNS	0.93 (0.84, 1.04)	0.93 (0.90, 0.97)	0.66 (0.46, 0.96)	0.65 (0.57, 0.75)	0.87 (0.75, 1.02)	0.81 (0.77, 0.86)	0.96 (0.72, 1.29)	0.92 (0.83, 1.02)	0.83 (0.63, 1.09)	0.80 (0.73, 0.88)
Chronic lower respiratory disease (including asthma)	0.87 (0.85, 0.90)	0.92 (0.91, 0.93)	0.78 (0.71, 0.86)	0.83 (0.80, 0.86)	0.85 (0.81, 0.88)	0.85 (0.84, 0.87)	0.98 (0.91, 1.06)	1.01 (0.98, 1.04)	0.88 (0.91, 1.05)	0.96 (0.94, 0.99)
Diseases of the digestive system	0.94 (0.91, 0.96)	0.96 (0.91, 0.97)	0.87 (0.80, 0.95)	0.90 (0.87, 0.92)	0.78 (0.74, 0.81)	0.76 (0.75, 0.77)	0.88 (0.81, 0.95)	0.89 (0.86, 0.91)	0.99 (0.93, 1.06)	1.00 (0.98, 1.03)
Diseases of the skin and soft tissue	1.06 (1.03, 1.09)	1.07 (1.06, 1.09)	1.24 (1.15, 1.33)	1.29 (1.25, 1.32)	1.36 (1.31, 1.41)	1.35 (1.33, 1.36)	1.24 (1.15, 1.33)	1.27 (1.24, 1.31)	1.26 (1.19, 1.34)	1.29 (1.26, 1.32)
Diseases of the musculoskeletal system and connective tissue	0.96 (0.92, 1.00)	0.98 (0.96, 0.99)	1.14 (1.03, 1.27)	1.13 (1.09, 1.18)	0.87 (0.82, 0.92)	0.85 (0.84, 0.87)	1.11 (1.00, 1.22)	1.14 (1.10, 1.18)	1.05 (0.96, 1.15)	1.05 (1.02, 1.09)
Urinary tract diseases	0.95 (0.89, 1.01)	0.98 (0.95, 1.00)	0.93 (0.77, 1.13)	0.96 (0.90, 1.02)	0.68 (0.61, 0.75)	0.66 (0.63, 0.68)	0.84 (0.69, 1.01)	0.83 (0.78, 0.89)	0.98 (0.84, 1.14)	1.00 (0.95, 1.05)
Genital diseases and disorders of breast	1.03 (0.98, 1.09)	1.05 (1.03, 1.06)	1.40 (1.23, 1.59)	1.40 (1.34, 1.47)	0.87 (0.81, 0.94)	0.86 (0.8, 0.88)	1.20 (1.06, 1.37)	1.20 (1.14, 1.25)	1.25 (1.12, 1.40)	1.23 (1.18, 1.28)
**Non-infectious neurological conditions**										
Neurological conditions total	0.99 (0.95, 1.03)	1.03 (1.01, 1.04)	1.00 (0.90, 1.12)	1.06 (1.02, 1.10)	0.81 (0.77, 0.86)	0.78 (0.76, 0.79)	1.08 (0.97, 1.20)	1.10 (1.06, 1.14)	1.11 (1.02, 1.22)	1.10 (1.07, 1.13)
Sleeping disorders	0.95 (0.89, 1.02)	1.02 (0.99, 1.05)	0.95 (0.79, 1.15)	0.99 (0.92, 1.07)	0.90 (0.82, 0.99)	0.90 (0.87, 0.93)	1.13 (0.95, 1.34)	1.18 (1.11, 1.26)	1.05 (0.90, 1.22)	1.00 (0.95, 1.06)
Neoplasms of the CNS (malignant and benign)	0.83 (0.55, 1.26)	0.85 (0.73, 0.98)	2.13 (1.01, 4.52)	1.86 (1.39, 2.48)	0.54 (0.27, 1.09)	0.53 (0.41, 0.69)	1.38 (0.57, 3.34)	1.32 (0.95, 1.81)	1.03 (0.43, 2.49)	1.24 (0.93, 0.88)
Cerebrovascular diseases	0.98 (0.68, 1.42)	0.93 (0.84, 1.04)	0.55 (0.14, 2.20)	0.66 (0.46, 0.96)	0.79 (0.46, 1.38)	0.87 (0.75, 1.02)	0.26 (0.52, 3.06)	0.96 (0.72, 1.29)	0.93 (0.39, 2.26)	0.83 (0.63, 1.09)
Epilepsy	0.99 (0.90, 1.09)	1.05 (1.02, 1.08)	1.04 (0.80, 1.36)	1.17 (1.07, 1.27)	0.87 (0.76, 1.00)	0.78 (0.74, 0.82)	0.98 (0.76, 1.27)	0.97 (0.86, 1.06)	1.18 (0.96, 1.45)	1.24 (1.16, 1.33)
Headache conditions (including migraine)	0.91 (0.84, 0.98)	0.94 (0.91, 0.96)	1.00 (0.81, 1.23)	1.03 (0.96, 1.10)	0.60 (0.53, 0.68)	0.59 (0.57, 0.61)	0.95 (0.78, 1.16)	0.98 (0.91, 1.04)	1.09 (0.92, 1.28)	1.07 (1.01, 1.13)
Cerebral palsy	0.91 (0.74, 1.12)	0.91 (0.84, 0.99)	1.37 (0.85, 2.21)	1.52 (1.28, 1.80)	0.85 (0.63, 1.13)	0.79 (0.70, 0.88)	0.68 (0.35, 1.30)	0.72 (0.57, 0.91)	0.99 (0.62, 1.58)	0.90 (0.75, 1.88)
Hydrocephalus	0.98 (0.73, 1.34)	0.90 (0.80, 1.02)	0.74 (0.27, 1.97)	0.81 (0.56, 1.17)	0.50 (0.28, 0.88)	0.55 (0.45, 0.68)	0.52 (0.17, 1.63)	0.48 (0.30, 0.75)	0.51 (0.19, 1.36)	0.53 (0.36, 0.76)
Other disorders of the nervous system	0.98 (0.68, 1.42)	1.16 (1.13, 1.19)	0.55 (0.14, 2.20)	1.23 (1.14, 1.32)	0.79 (0.46, 1.38)	0.95 (0.91, 0.98)	1.26 (0.52, 3.06)	1.35 (1.27, 1.44)	0.93 (0.39, 2.26)	1.38 (1.30, 1.45)

Odds ratio not calculated if *n*⩽5.

EU/EEA=EU/European Economic Area (EEA), Oceania, United States of America (USA) and Canada.

CNS: central nervous system.

### Infectious diseases

For infections in total and for five out of 11 specific diagnosis groups, children of one immigrant parent had lower hazards (in the range 2–22%) than children of Norwegian background. For three groups the hazard was higher (in the range 20–29% higher for infections of the skin and fungal and parasitic infections) (Table II). For tuberculosis, the number of cases was low in each group, but the hazard ratio high (6.35, 95% CI 3.53, 11.43). Children with an immigrant mother had lower hazards than children with Norwegian background of a diagnosis of any infectious condition (Table II). The hazard of infections in total was lower among children with a parent from EU/EEA and from Asia, but higher among children whose parent originated from Africa and Latin America (Table III).

### Non-infectious medical conditions

For non-infectious medical conditions in total and for nine of 18 of the specific conditions, children of one immigrant parent had lower hazards than children of Norwegian background (in the range 2–27% lower) (Table II). For five conditions, children of an immigrant parent had higher hazards than those with Norwegian background (in the range 4–17% higher). The highest hazards were seen for obesity and other nutritional deficiencies, and the lowest for immune system disorders and visual impairments. Those with an immigrant mother had lower hazards than children with Norwegian background of medical conditions in total and several specific conditions. Those with an immigrant father had higher hazards of some conditions (Table II). By parental region of origin, children with a parent from EU/EEA and Asia had lower, whereas children with a parent from Latin America higher, hazard of any medical condition (Table III). Children with parents from most regions of origin (except EU/EEA) had higher hazards for an obesity diagnosis than children of Norwegian background (highest for Africa: hazard ratio 2.23 (95% CI 1.76, 2.83)).

### Non-infectious neurological conditions

For most neurological conditions, the risk of a diagnosis did not vary between those with an immigrant parent and those with Norwegian background (Table II). For a diagnosis of any neurological condition children with one immigrant parent (and children with an immigrant mother) had lower hazards than children with Norwegian background. The hazards of the specific neurological conditions varied by whether the child had an immigrant mother or immigrant father. The risk of neurological conditions also varied over parental region of origin (Table III).

Adjustment for socioeconomic indicators did not explain differences in hazards of receiving diagnoses between children with one immigrant parent and with Norwegian background. For several conditions, differences were even more pronounced in adjusted models (Tables II and III).

## Discussion

### Main findings

The results did not indicate that the somatic health of children raised in exogamous families overall was worse than health among children raised with two Norwegian parents. The hazard of receiving a diagnosis of any somatic condition was lower among children with an immigrant mother and children with a parent from EU/EEA or Asia, and higher among children with an immigrant father or a parent from Europe except EU/EEA, Africa or Latin America compared with children with Norwegian background. There were, however, some differences in hazards of diagnoses of the various conditions. Socioeconomic indicators did generally not explain differences between groups.

### Comparison with previous research

Two previous studies, with slightly different age cut offs, have assessed the somatic health of children and youth raised in exogamous families in the Nordic countries [10,12]. In Denmark, the odds of being hospitalized were the same for Danish-born in exogamous families as in Danish background counterparts, adjusted for SEP [10]. Among Finnish children and youth, the risk of being treated for a somatic condition was slightly higher among those with an immigrant father, but not those with an immigrant mother, compared with Finnish background counterparts [12]. The results of both studies are in line with our findings.

### Immigrant mother versus immigrant father

With some exceptions, the hazards of a somatic diagnosis were higher among those with an immigrant father, but lower among those with an immigrant mother, compared with children with a Norwegian background. If lower hazards represent barriers to health care use, this may relate to mothers being the main agent for health care utilization. Such barriers can relate to both cultural perceptions of when to seek health care and obstacles reflecting poor host language proficiency or health literacy. Loi et al. [12] suggest that differences between those with an immigrant mother and an immigrant father lie in differences in which countries mothers and fathers often come from and how this translates into socioeconomic resources. This could have some relevance to our results as immigrant fathers most often originate from countries within the EU, whereas mothers most commonly come from Sweden, Philippines, Thailand and Denmark. This does not, however, explain why the hazards for some conditions were higher among children with immigrant fathers.

### Comparison with children with two immigrant parents

We have previously assessed differences in hazards for somatic diagnoses between children of two immigrant parents and children with Norwegian background, using the same dataset as in the present study [1]. Where children of two immigrant parents had lower hazards than Norwegian background counterparts, the hazards of children with one immigrant parent tended to be somewhere between those with two immigrant parents and those with two Norwegian-born parents. Those with an immigrant father were closer to those with two Norwegian-born parents than were those with an immigrant mother. This supports our hypothesis that barriers related to language and health care system knowledge play a role in health care seeking behaviour and risk of diagnoses among children of immigrant parents. Dang [17] found that an intensive integration and language programme improves the cumulative use of primary care services among refugees in Norway and has spillover benefits for the health of their children, suggesting a combinational effect of language, institutional knowledge, health literacy and improved social network.

Also, for diagnoses for which children of two immigrants had higher hazards than children with a Norwegian background, including obesity and some infectious diseases [1], children of one-immigrant parents generally had hazards between these two groups. Childhood obesity has a socioeconomic gradient [2], and a high prevalence of obesity among immigrant children has in other studies been partly explained by differences in behaviour related to nutrition and physical activity [2,18,19]. Although SEP has not shown to explain differences in obesity prevalence between children of immigrants and natives [2,20], higher SEP in exogamous families than in immigrant families may explain some of the differences in risk of an obesity diagnosis between children with one and children with two immigrant parents. The risk of infections among children of immigrants has been related to travels to parents’ country of origin, as well as to crowded households and number of siblings. Thus, these differences may also point towards the higher SEP and better living conditions of exogamous families compared with immigrants.

### Strengths and limitations

The study leverage register data with national coverage, and with linkage to sociodemographic variables from Statistics Norway. The quality of NPR in general is regarded to be high, although the completeness of a few diagnoses is shown to be somewhat lower than others [15]. Although registration errors in the NPR occur, such errors are likely to be fairly evenly distributed and not influenced by the patient’s or parents’ country of birth or background, and would have little impact on the relative relationship between the groups we have analysed. Our data include diagnoses given in specialist care only and may thus reflect more serious or further developed conditions than those treated in primary care. However, as some of the conditions could have been prevented if treated in a timely way in primary care, results might be influenced by differences in use of primary care. Moreover, the general practitioner is the gatekeeper to specialist health services in the Norwegian health care system. Data from primary care could thus have nuanced the picture described in this study. Importantly, differences in risk of receiving a diagnosis could reflect both differences in risk of a condition and in health care seeking behaviour. The included children were between 0 and 10 years old, and the differences in follow-up time between the children was handled by Cox regressions and adjustment for year of birth.

## Conclusions

Children of one immigrant parent do not have overall worse health than Norwegian background children, and it seems that they take a middle place between Norwegian background children and children with two immigrant parents in terms of somatic health conditions. Hazards of diagnoses of somatic health conditions were generally lower among those with an immigrant mother than among those with an immigrant father. This indicates a need to further assess whether these differences are related to underutilization of health care services and potential barriers to use. Differences between groups could not be explained by differences in socioeconomic factors.

## Supplemental Material

sj-docx-1-sjp-10.1177_14034948251344527 – Supplemental material for Diagnoses given in specialist health care to Norwegian-born children with one immigrant parent. A register-based study Supplemental material, sj-docx-1-sjp-10.1177_14034948251344527 for Diagnoses given in specialist health care to Norwegian-born children with one immigrant parent. A register-based study by Marte K. R. Kjøllesdal, Ylva Helland and Thor Indseth in Scandinavian Journal of Public Health
