# Supplementary material for: Diagnoses given in specialist health care to Norwegian-born children with one immigrant parent. A register-based study
Source: Scand J Public Health. 2025 Jun 12;54(6):572–80. doi: 10.1177/14034948251344527 (PMC13356262; doi:10.1177/14034948251344527)
Supplement: sj-docx-1-sjp-10.1177_14034948251344527 – Supplemental material for Diagnoses given in specialist health care to Norwegian-born children with one immigrant parent. A register-based study [file sj-docx-1-sjp-10.1177_14034948251344527.docx]

**Supplementary table 1.** Categorization of diseases

| **CATEGORY** | **ICD-10 CODES** |
| --- | --- |
| **Infections** |  |
| Intestinal infectious diseases | A00–A09 |
| Tuberculosis | A15–A19 |
| Other bacterial infections and sexually transmitted diseases | A20–A79, B95, B96, B98 |
| Viral infections | A80–B34, B97 |
| Fungal and parasitic infections | B35–B89 |
| Influenza and other acute lower respiratory tract infections | J09–J22 |
| Infections of the skin and subcutaneous tissue | L00–L08 |
| Infections of the musculoskeletal system and soft tissue^a^ | M00–M03, M60, M65, M86 |
| Urinary tract infections^a^ | N10, N12, N30, N34 |
| Genital infections^a^ | N41, N45, N49, N61, N70 – N77 |
| Infections of the CNS^b^ | G00–G09 |
| **Non-infectious medical conditions** |  |
| Malignant neoplasms outside of the CNS^b^ | C00–C69, C73–C97 |
| Benign neoplasms and carcinoma in situ, outside of the CNS^b^ | D00–D31, D34–D41, D44–D48 |
| Blood diseases | D50–D77 |
| Immune system disorders | D80–D89 |
| Endocrine disorders | E00–E35 |
| Malnutrition and problems with eating and feeding | E40–E46, R13, R63–R64 |
| Other nutritional deficiencies | E50–E64 |
| Obesity and other hyperalimentation | E65–E68 |
| Metabolic disorders | E70–E72, E74–E85, E88, E89 |
| Visual impairment/blindness | H54 |
| Hearing impairment/deafness | H90–H91 |
| Diseases of the circulatory system outside of the CNS^b, c^ | I00–I52, I70–I79, I81, I82 |
| Chronic lower respiratory disease (including asthma) | J40–J47 |
| Diseases of the digestive system^d^ | K20–K31, K40–K64, K70–K93 |
| Diseases of the skin and soft tissue | L10–L99 |
| Diseases of the musculoskeletal system and connective tissue | M05–M54, M61–M63, M66–M85, M87–M99 |
| Urinary tract diseases | N00–N08, N11, N13–N29, N31–N33, N35–N39 |
| Genital diseases and disorders of breast | N40, N42–N44, N46–N48, N50–N60, N62–N64, N80–N99 |
| **Non-infectious neurological conditions** |  |
| Sleeping disorders | G47, F51 |
| Neoplasms of the CNS^b^ (malignant and benign) | C70–C72, D32–D33, D42–D43 |
| Cerebrovascular diseases | I60–I69, G45, G46 |
| Epilepsy | G40–G41 |
| Headache conditions (including migraine) | G43–G44, R51 |
| Cerebral palsy | G80 |
| Hydrocephalus | G91 |
| Other disorders of the nervous system | G10–G37, G50–G73, G81–G90, G92–G99 |

^a^ Includes ICD-10 codes for inflammatory conditions that may also be caused by infections. ^b^ CNS = central nervous system.

^c^ Excluding venous disorders that mainly occur in elderly people. ^d^ Excluding disorders of the oral cavity, appendicitis and peritonitis/peritoneal disease.

| **Supplementary table 2.** Countries of origin in each region, sorted by number (highest-lowest) | | | | |
| --- | --- | --- | --- | --- |
| EU, EEA, Oceania, USA, Canada  N=76 966 | Europe outside EU/EEA  N=8290 | Asia  N=37 815 | Africa  N=9372 | Latin America  N=12 012 |
| \| Sweden \| \| --- \| \| Denmark \| \| Great Britain \| \| USA \| \| Germany \| \| Poland \| \| Netherland \| \| Finland \| \| France \| \| Iceland \| \| Canada \| \| Spain \| \| Australia \| \| Lithuania \| \| Romania \| \| Italy \| \| Switzerland \| \| Latvia \| \| Bulgaria \| \| Belgium \| \| Estonia \| \| Austria \| \| Ireland \| \| Croatia \| \| Hungary \| \| Portugal \| \| Greece \| \| New Zealand \| \| The Czech Republic \| \| The Faroe Islands \| \| Slovakia \| \| Greenland \| \| Luxembourg \| \| Slovenia \| \| Malta \| \| Fiji \| \| The Solomon Islands \| \| New-Caledonia \| \| Papa New Guinea \| \| French Polynesia \| \| Gibraltar \| \| Samoa \| \| Kiribati \| \| Tonga \| \| Monaco \| \| Palau \| \| Tuvalu \| \| Vanuatu \| \| Cook Islands \| \| American Samoa \| \| San Marino \| \| Liechtenstein \| | \| Kosovo \| \| --- \| \| Montenegro \| \| Serbia \| \| North-Macedonia \| \| Bosnia-Hercegovina \| \| Ukraine \| \| Russia \| \| Moldova \| \| Belarus \| \| Albania \| | \| Philippines \| \| --- \| \| Thailand \| \| Pakistan \| \| South Korea \| \| Turkey \| \| Iran \| \| Vietnam \| \| India \| \| Iraq \| \| China \| \| Sri Lanka \| \| Indonesia \| \| Japan \| \| Libanon \| \| Israel \| \| Singapore \| \| Hongkong \| \| Bangladesh \| \| Afghanistan \| \| Syria \| \| Malaysia \| \| Kazakhstan \| \| Nepal \| \| Taiwan \| \| Palestine \| \| Cambodia \| \| Kuwait \| \| Azerbaijan \| \| Jordan \| \| Usbekistan \| \| Kyrgyzstan \| \| Myanmar \| \| Cyprus \| \| Georgia \| \| Laos \| \| United Arab Emirates \| \| Mongolia \| \| Tajikistan \| \| Saudi-Arabia \| \| Armenia \| \| Bhutan \| \| Bangladesh \| \| North-Korea \| \| Brunei \| \| Jemen \| \| Oman \| \| Bahrain \| \| Turkmenistan \| \| East-Timor \| \| Qatar \| \| The Maldives \| | \| Marocco \| \| --- \| \| Kenya \| \| Ethiopia \| \| South-Africa \| \| Nigeria \| \| Somalia \| \| Ghana \| \| Tanzania \| \| Tunisia \| \| Gambia \| \| Uganda \| \| Egypt \| \| Cameroon \| \| Madagaskar \| \| Algerie \| \| Zambia \| \| Eritrea \| \| Congo \| \| Liberia \| \| Angola \| \| Ivory Coast \| \| Sierra Leone \| \| Zimbabwe \| \| Senegal \| \| Burundi \| \| Sudan \| \| Mozambique \| \| Cape Verde \| \| Botswana \| \| Rwanda \| \| Mauritius \| \| Libya \| \| Namibia \| \| Congo Brazzaville \| \| Guinea \| \| Niger \| \| Togo \| \| Malawi \| \| Mali \| \| Burkina Faso \| \| South Sudan \| \| Guinea-Bissau \| \| Mauritania \| \| The Seychelles \| \| Gabon \| \| West-Sahara \| \| Benin \| \| Eswatini \| \| St. Helena, Ascension and Tristan de Cunha \| \| Lesotho \| \| Central African Republic \| \| Tsjad \| | \| Brazil \| \| --- \| \| Chile \| \| Colombia \| \| Peru \| \| Mexico \| \| Cuba \| \| Argentina \| \| Ecuador \| \| Dominican Republic \| \| Guatemala \| \| Venezuela \| \| Bolivia \| \| Costa Rica \| \| Trinidad and Tobago \| \| Jamaica \| \| Nicaragua \| \| Uruguay \| \| Paraguay \| \| El Salvador \| \| Honduras \| \| Panama \| \| Guyana \| \| Haiti \| \| Curacao \| \| Puerto Rico \| \| Surinam \| \| Belize \| \| Bahamas \| \| Grenada \| \| Barbados \| \| Dominica \| \| St. Lucia \| \| Antigua and Barbuda \| \| Bermuda \| \| Martinique \| \| The US Virgin Islands \| \| The British Virgin Islands \| \| Aruba \| \| St.Vincent and the Grenadines \| \| Cayman Islands \| \| Guadeloupe \| \| French Guyana \| |

| **Supplementary table 3a.** Number (per 1000) with diagnosis given in secondary and tertiary health care between 2008 and 2018 among Norwegian-born children 0-10 year having two Norwegian-born parents or one immigrant parent. | | | | |
| --- | --- | --- | --- | --- |
|  | Norwegian background  N=846 182 | Norwegian-born to one immigrant parent, total  N=144 455 | Norwegian-born to immigrant mother  N=78 497 | Norwegian-born to immigrant father  N=65 958 |
| **Any somatic condition** | 306 224 (361.9) | 54 029 (374.0)*** | 29 173 (371.6)*** | 24 856 (376.8)*** |
| **Infections** |  |  |  |  |
| Infections total | 110 815 (130.1) | 19 978 (138.3)*** | 10 638 (135.5)*** | 9340 (141.6)*** |
| Intestinal infectious diseases | 25 878 (30.6) | 4806 (33.3)*** | 2606 (33.2)*** | 2200 (33.4)*** |
| Tuberculosis | 27 (0.0) | 34 (0.2)*** | 26 (0.3)*** | 8 (0.1)**** |
| Other bacterial infections and sexually transmitted diseases | 8917 (10.5) | 1517 (10.5) | 826 (10.5) | 691 (10.5) |
| Viral infections | 27 531 (32.5) | 4848 (33.6)* | 2563 (32.7) | 2285 (34.6)** |
| Fungal and parasitic infections | 2676 (3.2) | 584 (4.0)*** | 315 (4.0)*** | 269 (4.1)*** |
| Influenza and other acute lower respiratory tract infections | 44 260 (52.3) | 7237 (50.1)*** | 3713 (47.3)*** | 3524 (53.4) |
| Infections of the skin and subcutaneous tissue | 12 098 (14.3) | 2875 (19.9)*** | 1579 (20.1)*** | 1296 (19.6)*** |
| Infections of the musculoskeletal system and soft tissue | 5269 (6.2) | 854 (5.9) | 462 (5.9) | 392 (5.9) |
| Urinary tract infections | 7791 (9.2) | 1385 (9.6) | 743 (9.5) | 642 (9.7) |
| Genital Infections | 1590 (1.9) | 283 (2.0) | 158 (2.0) | 125 (1.9) |
| Infections of the CNS | 1541 (1.8) | 229 (1.6) | 104 (1.3)** | 125 (1.9) |
| **Non-infectious medical conditions** |  |  |  |  |
| Any non-infectious medical condition | 242 944 (287.1) | 42626 (295.1)*** | 23 066 (293.8)*** | 19 560 (296.6)*** |
| Malignant neoplasms (outside CNS) | 1197 (1.4) | 198 (1.4) | 103 (1.3) | 95 (1.4) |
| Benign neoplasms (outside CNS) | 18 397 (21.7) | 3232 (22.4) | 1761 (22.4) | 1471 (22.2) |
| Blood diseases | 6946 (8.2) | 1360 (9.4)*** | 724 (9.2)** | 636 (9.6)*** |
| Immune system disorders | 997 (1.2) | 147 (1.0) | 80 (1.0) | 67 (1.0) |
| Endocrine disorders | 9505 (11.2) | 1490 (10.3)** | 749 (9.5)*** | 741 (11.2) |
| Malnutrition and problems with eating and feeding | 10 560 (12.5) | 2190 (15.2)*** | 1215 (15.5)*** | 975 (14.8)*** |
| Other nutritional deficiencies | 1766 (2.1) | 396 (2.7)*** | 196 (2.5)* | 200 (3.0)*** |
| Obesity and other hyperalimentation | 3367 (4.0) | 647 (4.5)** | 304 (3.9) | 343 (5.2)*** |
| Metabolic disorders | 2282 (2.7) | 324 (2.2)** | 166 (2.1)** | 158 (2.4) |
| Visual impairment/blindness | 1147 (1.4) | 146 (1.0)** | 73 (0.9)** | 73 (1.1) |
| Hearing impairment/deafness | 17 368 (20.5) | 2602 (18.0)*** | 1416 (18.0)*** | 1186 (18.0)*** |
| Diseases of the circulatory system outside of the CNS | 6041 (7.1) | 956 (6.6)* | 521 (6.6) | 435 (6.6) |
| Chronic lower respiratory disease (including asthma) | 64 363 (76.1) | 10 060 (69.6)*** | 5291 (67.4)*** | 4769 (72.3)*** |
| Diseases of the digestive system | 71 802 (84.9) | 11 862 (82.1)*** | 6123 (78.0)*** | 5739 (87.0) |
| Diseases of the skin and soft tissue | 62594 (74.0) | 13 726 (95.0)*** | 7936 (101.1)*** | 5790 (87.8)*** |
| Diseases of the musculoskeletal system and connective tissue | 34 600 (40.9) | 5660 (39.2)** | 2918 (37.2)*** | 2742 (41.6) |
| Urinary tract diseases | 14 310 (16.9) | 2139 (14.8)*** | 1128 (14.4)*** | 1011 (15.3)** |
| Gential diseases and disorders of breast | 20 065 (23.7) | 3655 (25.3)*** | 1939 (24.7) | 1716 (26.0)*** |
| **Non-infectious neurological conditions** |  |  |  |  |
| Neurological conditions total | 36 060 (42.6) | 5974 (41.4)* | 3063 (39.0)*** | 2911 (44.1) |
| Sleeping disorders | 10 241 (12.1) | 1851 (12.8)* | 929 (11.8) | 922 (14.0)*** |
| Neoplasms of the CNS (malignant and benign) | 441 (0.5) | 64 (0.4) | 38 (0.5) | 26 (0.4) |
| Cerebrovascular diseases | 594 (0.7) | 103 (0.7) | 54 (0.7) | 49 (0.7)* |
| Epilepsy | 7372 (8.7) | 1163 (8.1)** | 592 (7.5) | 571 (8.7)*** |
| Headache conditions (including migraine) | 10 417 (12.3) | 1428 (9.9)*** | 715 (9.1)*** | 713 (10.8)** |
| Cerebral palsy | 2139 (2.5) | 308 (2.1)* | 163 (2.1)* | 145 (2.2) |
| Hydrocephalus | 995 (1.2) | 151 (1.0) | 83 (1.1) | 68 (1.0) |
| Other disorders of the nervous system | 10 032 (11.9) | 1953 (13.5)*** | 1024 (13.0)** | 929 (14.1)*** |
| Difference to Norwegian background children for each region and diagnosis. ***P<0.001, **p<0.01, *p<0.05. From Chi square tests | | | | |

| **Supplementary table 3b.** Number (per 1000) with diagnosis given in secondary and tertiary health care between 2008 and 2018 among Norwegian-born children 0-10 year having one immigrant parent, by parental region of origin. | | | | | |
| --- | --- | --- | --- | --- | --- |
|  | EU, EEA, Oceania, USA, Canada  N= 76 966 | Europe except EU/EEA  N= 8290 | Asia  N= 37 815 | Africa  N= 9372 | Latin America  N=12 012 |
| **Any somatic condition** | 27 793 (361.1)*** | 3339 (402.8)*** | 14 160 (374.5)*** | 3727 (397.7)*** | 5010 (417.1)*** |
| **Infections** |  |  |  |  |  |
| Infections total | 10 071 (130.8)*** | 1253 (151.1) | 5231 (138.3)*** | 1460 (155.8) | 1963 (163.4) |
| Intestinal infectious diseases | 2282 (29.6) | 292 (35.2)* | 1320 (34.9)*** | 351 (37.5)*** | 561 (46.7)*** |
| Tuberculosis | - | - | 17 (0.4) | - | - |
| Other bacterial infections and sexually transmitted diseases | 786 (10.2) | 88 (10.6) | 427 (11.3) | 89 (9.5) | 127 (10.6) |
| Viral infections | 2528 (32.8) | 281 (33.9) | 1217 (32.2) | 341 (36.4) | 481 (40.0) |
| Fungal and parasitic infections | 257 (3.3) | 36 (4.3) | 153 (4.0)** | 70 (7.5)*** | 68 (5.7)*** |
| Influenza and other acute lower respiratory tract infections | 3679 (47.8)*** | 403 (48.6) | 1962 (51.9) | 512 (44.6) | 681 (56.7)* |
| Infections of the skin and subcutaneous tissue | 1352 (17.6)*** | 195 (23.5)*** | 780 (20.6)*** | 252 (28.0)*** | 286 (23.8)*** |
| Infections of the musculoskeletal system and soft tissue | 459 (6.0) | 53 (6.4) | 209 (5.5) | 51 (5.4) | 82 (6.8) |
| Urinary tract infections | 725 (9.4) | 98 (11.8) | 346 (9.4) | 90 (9.6) | 126 (10.5) |
| Genital Infections | 134 (1.7) | 22 (2.7) | 79 (2.1) | 21 (2.2) | 27 (2.2) |
| Infections of the CNS | 136 (1.8) | 16 (1.9) | 50 (1.3)* | 14 (1.5) | 14 (1.2) |
| **Non-infectious medical conditions** |  |  |  |  |  |
| Any non-infectious medical condition | 21 858 (284.0) | 2635 (317.9)*** | 11 341 (299.9)*** | 2897 (309.1)*** | 3895 (324.3)*** |
| Malignant neoplasms (outside CNS) | 102 (1.3) | 11 (1.3) | 46 (1.2) | 19 (2.0) | 20 (1.7) |
| Benign neoplasms (outside CNS) | 1694 (22.0) | 223 (26.9)** | 832 (22.0) | 175 (18.7)* | 308 (25.6)** |
| Blood diseases | 636 (8.3) | 72 (8.7) | 445 (11.8)*** | 105 (11.2)** | 102 (8.5) |
| Immune system disorders | 82 (1.1) | 4 (0.5) | 39 (1.0) | 11 (1.2) | 11 (0.9) |
| Endocrine disorders | 803 (10.4)* | 74 (8.9)* | 371 (9.8)* | 119 (12.7) | 123 (10.2) |
| Malnutrition and problems with eating and feeding | 1040 (13.5)* | 116 (14.0) | 698 (18.5)*** | 138 (14.7) | 198 (16.5)*** |
| Other nutritional deficiencies | 156 (2.0) | 26 (3.1)* | 159 (4.2)*** | 26 (2.8) | 29 (2.4) |
| Obesity and other hyperalimentation | 242 (3.1)*** | 51 (6.2)** | 207 (5.5)*** | 81 (8.6)*** | 66 (5.5)** |
| Metabolic disorders | 176 (2.3)* | 23 (2.8) | 85 (2.2) | 18 (1.9) | 22 (1.8) |
| Visual impairment/blindness | 82 (1.1)* | 6 (0.7) | 44 (1.2) | 7 (0.7) | 7 (0.6)* |
| Hearing impairment/deafness | 1399 (18.2)*** | 144 (17.4)* | 654 (17.3)*** | 160 (17.1)* | 245 (20.4) |
| Diseases of the circulatory system outside of the CNS | 517 (6.7)*** | 40 (4.8)* | 249 (6.6) | 73 (7.8) | 77 (6.4) |
| Chronic lower respiratory disease (including asthma) | 5200 (67.6)*** | 532 (64.2)*** | 2624 (69.4)*** | 732 (78.1) | 972 (80.9)* |
| Diseases of the digestive system | 6448 (83.8) | 714 (86.1) | 2832 (74.9)*** | 763 (81.4) | 1105 (92.0)* |
| Diseases of the skin and soft tissue | 6245 (81.1)*** | 875 (105.5)*** | 4382 (115.9)*** | 924 (98.6)*** | 1300 (108.2)*** |
| Diseases of the musculoskeletal system and connective tissue | 2976 (38.7)** | 380 (45.8)* | 1347 (35.6)*** | 427 (55.6)* | 530 (44.1) |
| Urinary tract diseases | 1216 (15.8)* | 138 (16.6) | 457 (12.1)*** | 131 (14.0)* | 197 (16.4) |
| Gential diseases and disorders of breast | 1878 (24.4) | 296 (35.7)*** | 834 (22.1)* | 281 (30.0)*** | 366 (30.5)*** |
| **Non-infectious neurological conditions** |  |  |  | . |  |
| Neurological conditions total | 3213 (41.7) | 368 (44.4) | 1341 (35.5)*** | 445 (47.5)* | 607 (50.5)*** |
| Sleeping disorders | 933 (12.1) | 116 (14.0) | 475 (12.6) | 148 (15.8)** | 179 (14.9)** |
| Neoplasms of the CNS (malignant and benign) | 32 (0.4) | 8 (1.0) | 10 (0.3)* | 8 (0.9) | 8 (0.5) |
| Cerebrovascular diseases | 61 (0.8) | 5 (0.6) | 21 (0.6) | 8 (0.9) | 6 (0.7) |
| Epilepsy | 636 (8.3) | 70 (8.4) | 257 (6.8)*** | 81 (8.6) | 119 (9.9) |
| Headache conditions (including migraine) | 813 (10.6)*** | 93 (11.2) | 263 (7.0)*** | 107 (11.4) | 152 (12.7) |
| Cerebral palsy | 163 (2.1)* | 22 (2.7) | 76 (2.0)* | 21 (2.2)* | 26 (2.2) |
| Hydrocephalus | 95 (1.2)* | 10 (1.2) | 27 (0.7)* | 6 (0.6)* | 13 (1.1) |
| Other disorders of the nervous system | 1014 (13.2)** | 121 (14.6)* | 456 (12.1) | 149 (15.9)*** | 213 (17.7)*** |
| Difference to Norwegian background children for each region and diagnosis . ***P<0.001, **p<0.01, *p<0.05. From Chi square tests  N≤5 not shown | | | | | |
